# MiR-155 Induction by *F. novicida* but Not the Virulent *F. tularensis* Results in SHIP Down-Regulation and Enhanced Pro-Inflammatory Cytokine Response

**DOI:** 10.1371/journal.pone.0008508

**Published:** 2009-12-30

**Authors:** Thomas J. Cremer, David H. Ravneberg, Corey D. Clay, Melissa G. Piper-Hunter, Clay B. Marsh, Terry S. Elton, John S. Gunn, Amal Amer, Thirumala-Devi Kanneganti, Larry S. Schlesinger, Jonathan P. Butchar, Susheela Tridandapani

**Affiliations:** 1 Molecular, Cellular, and Developmental Biology Program, The Ohio State University, Columbus, Ohio, United States of America; 2 Integrated Biomedical Science Graduate Program, The Ohio State University, Columbus, Ohio, United States of America; 3 Department of Internal Medicine, The Ohio State University, Columbus, Ohio, United States of America; 4 College of Pharmacy, The Ohio State University, Columbus, Ohio, United States of America; 5 Center for Microbial Interface Biology, The Ohio State University, Columbus, Ohio, United States of America; 6 St. Jude Children's Research Hospital, Memphis, Tennessee, United States of America; University of Birmingham, United Kingdom

## Abstract

The intracellular Gram-negative bacterium *Francisella tularensis* causes the disease tularemia and is known for its ability to subvert host immune responses. Previous work from our laboratory identified the PI3K/Akt pathway and SHIP as critical modulators of host resistance to *Francisella*. Here, we show that SHIP expression is strongly down-regulated in monocytes and macrophages following infection with *F. tularensis novicida* (*F.n.*). To account for this negative regulation we explored the possibility that microRNAs (miRs) that target SHIP may be induced during infection. There is one miR that is predicted to target SHIP, miR-155. We tested for induction and found that *F.n.* induced miR-155 both in primary monocytes/macrophages and *in vivo*. Using luciferase reporter assays we confirmed that miR-155 led to down-regulation of SHIP, showing that it specifically targets the SHIP 3′UTR. Further experiments showed that miR-155 and *BIC*, the gene that encodes miR-155, were induced as early as four hours post-infection in primary human monocytes. This expression was dependent on TLR2/MyD88 and did not require inflammasome activation. Importantly, miR-155 positively regulated pro-inflammatory cytokine release in human monocytes infected with *Francisella*. In sharp contrast, we found that the highly virulent type A SCHU S4 strain of *Francisella tularensis* (*F.t.*) led to a significantly lower miR-155 response than the less virulent *F.n.* Hence, *F.n.* induces miR-155 expression and leads to down-regulation of SHIP, resulting in enhanced pro-inflammatory responses. However, impaired miR-155 induction by SCHU S4 may help explain the lack of both SHIP down-regulation and pro-inflammatory response and may account for the virulence of Type A *Francisella*.

## Introduction

*Francisella tularensis* is a highly infectious Gram-negative bacterium that infects phagocytic cells of the immune system [1]–[3]. Exposure to this pathogen causes the disease known as tularemia and a dose of as few as ten colony forming units can be lethal to humans [4], [5]. Thus, the CDC has classified this pathogen as a Category A select agent [6]. There are five known subspecies of *Francisella* [5], [7], and the subspecies *novicida* (*F.n.*) is less infectious to humans than the Type A *F. tularensis* subspecies *tularensis* (*F.t.*). However, *F.n.* provides an excellent model organism because it shares a similar intracellular lifecycle to *F.t.* and leads to tularemia-like pathologies in mouse models [4].

Effective host cell defense against *Francisella* is subverted by numerous mechanisms. Indeed, interferon response [8], toll-like receptor (TLR) signaling [9]–[11], and antigen presentation [11], [12] are all found to be compromised by *Francisella*. Although Gram-negative, *Francisella* presents a modified form of lipopolysaccharide (LPS) that only minimally activates TLR4 [10], [13]. Instead, most of the cell surface-driven host response is through TLR2 [14], [15] and finding ways to enhance the response are of interest for novel therapeutics [16]. Understanding these host responses and how *Francisella* undermines them is critical for our ability to successfully prevent and treat infection.

One important response downstream of TLR stimulation is activation of the PI3K/Akt pathway [17], [18]. Our laboratory has shown that PI3K/Akt activation is host-protective against *Francisella*, as mice expressing a macrophage-specific, constitutively-active form of Akt are protected from an otherwise-lethal challenge compared to wild-type littermates [18]. Expectedly, the PI3K/Akt pathway is subject to negative regulation and one of the key regulators is SHIP, which we have also found to be important during *Franciesella* infection. Murine bone marrow-derived macrophages (BMM) lacking SHIP display increased NFκB activity and enhanced cytokine production [17], similar to the responses seen with constitutively active Akt [18]. More recently we have found that the loss of SHIP or constitutive activation of Akt promotes phagosome-lysosome fusion of *Francisella* in macrophages. Thus this pathway is important for intracellular control [19], [20]. Collectively, these findings implicate the PI3K/Akt pathway and SHIP as critical regulators of the host-response to *Francisella*.

While studying the role of SHIP during *Francisella* infection, we found that infection with the less virulent *F.n.* leads to SHIP down-regulation. In a search for mechanisms to explain this, we entertained the possibility that microRNAs may play a role. In fact during the preparation of this manuscript it was reported that SHIP is targeted by a microRNA, miR-155 [21]. MiRs are a recently-discovered means of regulating both transcript and protein levels of specific genes [22]. They are processed yet non-translated RNA molecules of approximately 22 base pairs in length that primarily target the 3′untranslated region (UTR) of mRNA transcripts in a sequence-specific manner. Recent reports show that treatment with TLR ligands can induce miR-146 and miR-155 expression within macrophages [23], [24]. Yet the significance of these findings within the context of a bacterial infection remains largely unknown; however, is of great interest [20].

Here, we show that *Francisella* infection induces miR-155 expression in a TLR-dependent manner and that this leads to down-regulation of SHIP. Notably, the less virulent *F.n.* subspecies strongly induces miR-155 while the virulent *F.t.* isolate SCHU S4 does not. Hence, expression of miR-155 is a component of host defense against *Francisella* and the difference in miR-155 response to these two subspecies may help explain the success of *F.t.* as an infectious agent in humans.

## Results

### SHIP Expression Is Down-Regulated in Response to *F.n.* Infection

**Figure 1:**
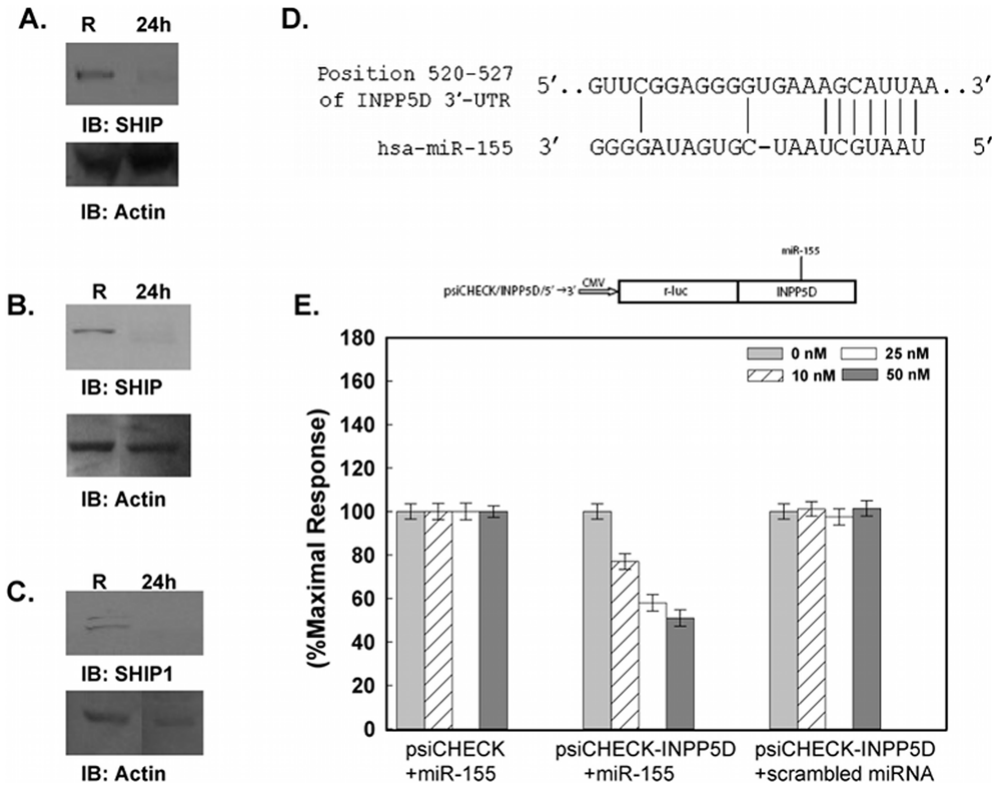
SHIP expression is down-regulated in response to *F.n.* infection. (A) PBM were infected with *F.n.* at an MOI of 50 for 24 hours. Cell lysates were analyzed by Western blotting use an anti-SHIP antibody in the top panel. ‘R’ designates resting/uninfected cells and ‘24 h’ designates infected with *F.n.* The lower panel is a reprobe of the same membrane with an anti-actin antibody. (B) THP-1 were infected with *F.n.* at an MOI of 100 for 24 hours. Cell lysates were resolved as in Figure 1A. (C) Western blots for SHIP in murine bone marrow-derived macrophages infected with *F.n.* at an MOI of 50 for 24 hours. Cell lysates were resolved as in Figure 1A. (D) Predicted interaction between miR-155 and the 3′UTR of SHIP (INPP5D) mRNA. (E) Normalized luciferase activity in cells transfected with the 3′UTR of SHIP (psiCHECK_INPP5D) or with vector alone (psiCHECK), and cotransfected with a control *Renilla* luciferase vector. Synthetic miR-155 or non-specific (scrambled) miRs were subsequently transfected at concentrations of 0, 10, 25 and 50 nM. Luminometer readings were taken 48 hours post-transfection. The graph represents *f-luc* expression normalized to *r-luc* expression, then normalized to percent maximal response.

We have previously demonstrated that the pro-inflammatory cytokine response to *Francisella* infection requires the activation of the PI3K/Akt pathway and that this response is down-regulated by SHIP, an established negative regulator of the PI3K/Akt pathway [17], [18]. To follow up these findings, we examined the expression of SHIP during *F.n.* infection. Here, human PBM were infected with *F.n.* (MOI 50) for 24 hours and SHIP protein levels were analyzed by Western blotting. Results, shown in Figure 1A, demonstrate a dramatic down-regulation of SHIP in infected cells versus uninfected controls. The lower panel is a reprobe of the same membrane with actin antibody to ensure equal loading of protein in both lanes. Of note, viability of PBM at 24 hours post infection was greater than 90%. Similar down-regulation of SHIP was also found in the human monocytic cell line THP-1 (Figure 1B), as well as in primary murine bone marrow-derived macrophages (BMM) (Figure 1C) infected with *F.n.* for 24 hours at an MOI of 100 and 50, respectively.

In a search for potential mechanisms responsible for this down-regulation, we explored the possibility that microRNAs (miRs) may be involved. We used the online bioinformatics program TargetScan (http://www.targetscan.org) to identify potential miRs that may regulate SHIP. The program identified miR-155 as the sole predicted miR to bind SHIP on its 3′ UTR (Figure 1D). To assess the ability of miR-155 to specifically target the 3′UTR of SHIP we performed dual-luciferase reporter assays. For this, an *f-luc* luciferase reporter alone (psiCHECK) or the same reporter with the 3′UTR of SHIP (psiCHECK-INPP5D) were separately transfected into chinese hamster ovary (CHO) cells using Lipofectamine 2000. To control for differences in transfection efficiency a pRL-CMV *Renilla* luciferase construct was co-transfected with the psiCHECK vectors. To test the effect of miR-155 on expression of the transfected SHIP 3′ UTR reporter, synthetic miR-155 or scrambled miR were co-transfected into the cells at concentrations ranging from 10 nM to 50 nM. At 48 hours post-transfection cells were washed and lysed. Dual-luciferase activity in the transfectants was then measured. As shown in Figure 1E, the introduction of miR-155 suppressed the luciferase activity of psiCHECK-INPP5D but not of psiCHECK alone, indicating that miR-155 targeted the 3′ UTR of SHIP. As an additional control, cells were transfected with psiCHECK-INPP5D followed by a scrambled miR, and these showed no decrease in luciferase production.

### MiR-155 Is Induced In Vitro and In Vivo in Response to *F.n.* Infection

**Figure 2:**
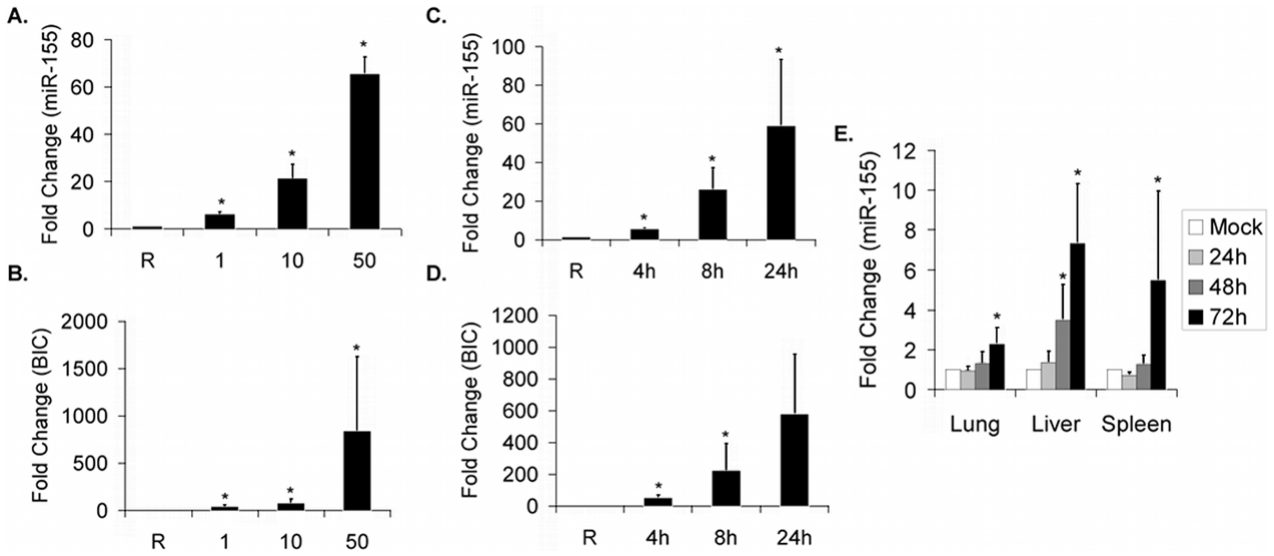
miR-155 is induced *in vitro* and *in vivo* in response to *F.n.* infection. (A–B) PBM were infected with *F.n.* for 24 hours at an MOI of 1, 10, or 50. Relative miR-155 (A) and *BIC* (B) expression were measured by qRT-PCR and then converted to fold change over uninfected. (C–D) PBM were infected with *F.n.* at an MOI of 50 for 4, 8, or 24 hours. Relative miR-155 (C) and *BIC* (D) expression were measured by qRT-PCR and then converted to fold change over uninfected. Graphs represent the mean and standard deviation of samples from three independent infections. (E) Mice were injected peritoneally with 200 CFU of *F.n.* or PBS, then euthanized at 24, 48, and 72 hours post-infection. Relative miR-155 expression was measured by qRT-PCR for the liver, lung and spleen and then converted to fold change over uninfected. An *n* of 6 was used for each timepoint. Graphs represent the mean and standard deviation. Data were analyzed by a paired Student *t* test. An asterisk (*) indicates a *p*-value<0.05.

Having established that SHIP is down-regulated during *F.n.* infection and that miR-155 is a negative regulator of SHIP, we next examined whether mature miR-155 and *BIC*, the non-protein coding gene that encodes miR-155 [25], mRNA were induced in infected cells. For this, human PBM were infected with *F.n.* and the expression of miR-155 and *BIC* were measured by qRT-PCR. Results showed a dose-dependent induction of mature miR-155 (Figure 2A) as well as *BIC* (Figure 2B) in PBM infected at MOI 1, 10, or 50 for 24 hours. We next examined the time course of miR-155 and *BIC* induction. Results showed a gradual increase in both mature miR-155 (Figure 2C) and *BIC* mRNA (Figure 2D) from 4 to 24 hours post-infection when infected at an MOI of 50. This time course of mature miR-155 induction is consistent with previous findings in murine macrophages stimulated with toll-like receptor ligands [24].

Next, we tested whether *F.n.* infection led to miR-155 induction *in vivo*. For this, mice were challenged with 200 CFU of *F.n.* or with PBS, delivered intraperitoneally. The animals were sacrificed at 24, 48, and 72 hours post-infection (n = 6 per time point) and the lungs, liver, and spleen were harvested. RNA from homogenates was assayed for mature miR-155 by qRT-PCR (Figure 2E). Tissue-specific patterns of miR-155 matched those previously reported [26], where basal relative expression was highest in the spleen and lowest in the liver. Results showed a clear *F.n.*-induced induction of miR-155 in all organs tested, suggesting that *F.n.* elicits expression of mature miR-155 *in vivo*.

### Bacterial Viability Contributes to MiR-155 Induction

**Figure 3:**
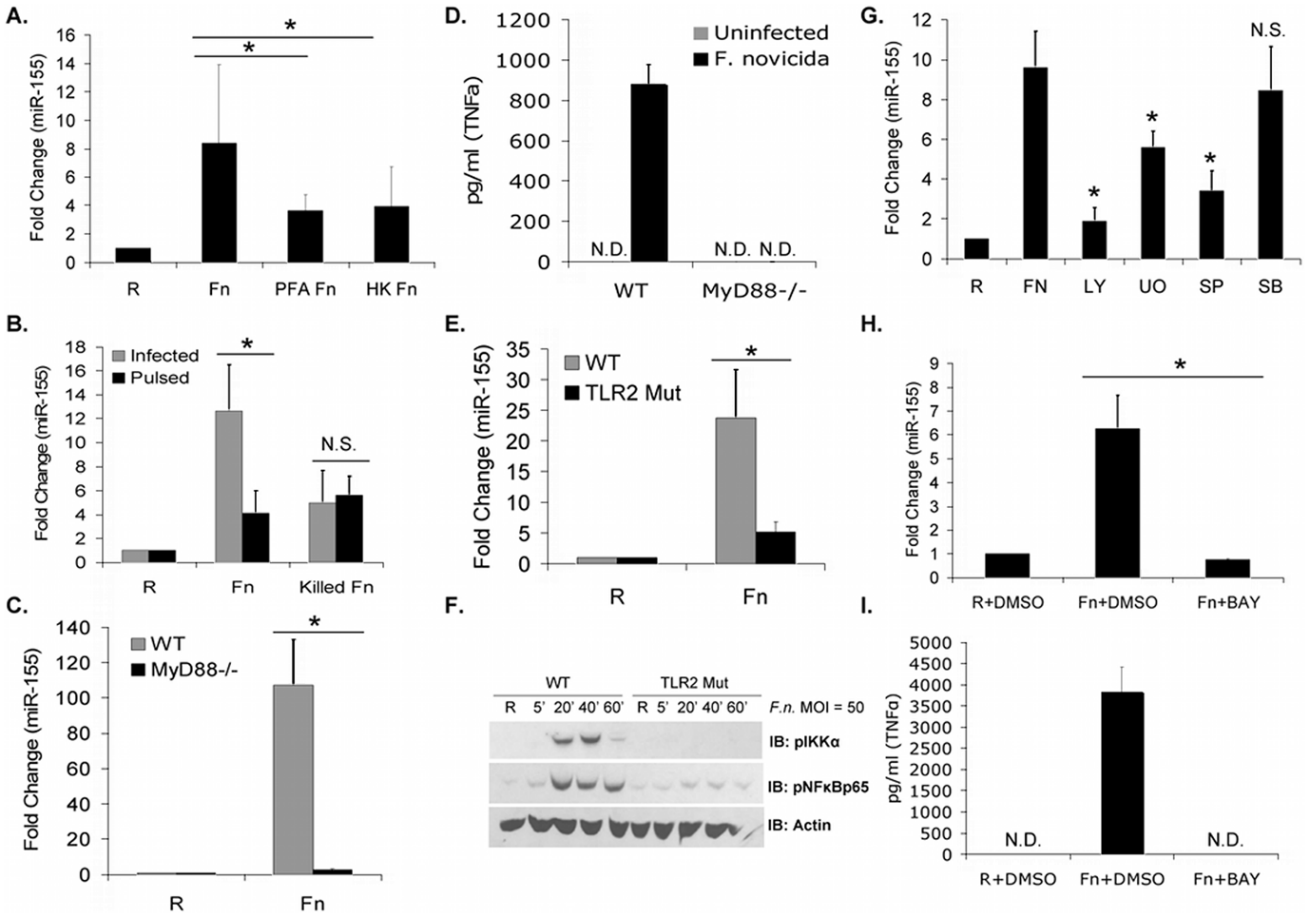
Bacterial viability contributes to miR-155 induction through the TLR pathway. (A) PBM were infected with live, heat-killed (HK), or paraformaldehyde-fixed (PFA) *F.n.* at an MOI of 50 for 24 hours, then miR-155 measured by qRT-PCR. (B) PBM were infected with live *F.n.* or heat-killed *F.n.* at an MOI of 50, then with or without gentamicin after 2 hours, then miR-155 was assayed by qRT-PCR. (C) Wild-type (WT) or MyD88^−/−^ bone marrow-derived macrophages were infected with *F.n.* at an MOI of 50 for 24 hours, then relative miR-155 expression measured by qRT-PCR and then converted to fold change over uninfected. (D) ELISA for TNFα secretion in supernatants from the samples in Figure 3C. (E) Wild-type (WT) and TLR2 (TLR2 Mut) signaling mutant bone marrow-derived macrophages were infected with *F.n.* at an MOI of 50 for eight hours and miR-155 was assayed by qRT-PCR. (F) WT and TLR2 Mut macrophages were infected at an MOI of 50 of *F.n.* for 5, 20, 40, and 60 minutes. Cell lysates were analyzed by Western blotting for pIKKα, pNFκBp65, and Actin. (G) PBM were pretreated for 30 minutes with inhibitors of PI3K (LY), ERK (UO), JNK (SP), p38 (SB), or with DMSO vehicle control, infected with *F.n.* at an MOI of 50 for 6 hours, then miR-155 was assayed by qRT-PCR. (H) PBM were pretreated with the IKK inhibitor BAY7085 (BAY) or DMSO vehicle control for 90 minutes, infected with *F.n.* at an MOI of 50 for 6 hours, then miR-155 was assayed by qRT-PCR. (I) ELISA for TNFα in supernatants from the samples in Figure 3F. All experiments were performed in triplicate, and each experiment performed three times. Graphs represent mean and standard deviation. Data were analyzed by a paired Student *t* test. An asterisk (*) indicates a *p*-value<0.05.

Since infection with *F.n.* induces miR-155, we next tested whether bacterial viability was required for this response. PBM were infected with live, paraformaldehyde-killed or heat-killed *F.n.* at an MOI of 50 for 24 hours. While both forms of killed bacteria were able to induce relative miR-155 levels above that of uninfected cells, the levels were lower than what is induced by live bacteria (Figure 3A). To gain a better understanding of why viability is important, we examined miR-155 levels after 24 hours in PBM either exposed to 50 MOI of *F.n.* for the full 24 hour period or exposed for 2 hours before removal of extracellular bacteria. The PBM exposed to *F.n.* for the full 24 hours showed a stronger induction in miR-155 than those exposed for only 2 hours (Figure 3B). Conversely, levels of miR-155 induced by killed bacteria were comparable between the continuous exposure and the 2-hour exposure. These results suggest that a single exposure to *F.n.* induces miR-155, but that continuous infection elicits higher levels. These results also help explain Figures 2C and 2D, where the highest level of miR-155 was seen at 24 hours.

### MiR-155 Induction by *F.n.* Requires Activation of the TLR Signaling Pathway

Previous studies have determined that TLR2 is critical for the recognition of *Francisella* and induction of the pro-inflammatory response. To test the involvement of TLR signaling in miR-155 induction we infected bone marrow-derived macrophages (BMM) from either wild-type or MyD88^−/−^ mice with *F.n.* at an MOI of 50 for 24 hours. Wild-type macrophages showed strong induction of miR-155, whereas the miR-155 response in MyD88^−/−^ macrophages was drastically reduced (Figure 3C). Confirming the absence of MyD88, we found that TNFα was not secreted by the MyD88^−/−^ macrophages following infection (Figure 3D) as should be expected given that TNFα induction is entirely TLR2- and MyD88-dependent [14]. Hence, miR-155 induction by *F.n.* is MyD88-dependent.

To further test the role of TLR signaling in the induction of miR-155 we infected wild-type and TLR2 signaling mutant macrophages with *F.n*. at an MOI of 50 for eight hours. The results show that TLR2 is required for the induction of miR-155 (Figure 3E). As a control for the TLR2 signaling mutant we tested for signaling activation of the NFκB pathway. It is clear that the TLR2 signaling mutant functions as expected since these macrophages do not display activation of the IKK complex or lead to NFκBp65 phosphorylation whereas the wild-type do (Figure 3F).

We next tested the involvement of PI3K and the mitogen-activated protein kinases (MAPKs), downstream mediators known to be activated during *Francisella* infection [17]. PBM were pretreated with LY294002 (PI3K inhibitor), U0126 (ERK inhibitor), SP00125 (JNK inhibitor), SB203580 (p38 inhibitor) or DMSO vehicle control for 30 minutes and then infected with *F.n.* for 6 hours at an MOI of 50. Inhibition of PI3K completely blocked miR-155 induction (Figure 3G). An earlier report in B cells showed that the MAPKs ERK and JNK but not p38 were necessary for miR-155 induction by B-cell receptor activation [27]. Consistent with this, we found that inhibition of both ERK and JNK resulted in reduced miR-155 induction but inhibition of p38 showed no effect. Given these results we can conclude that miR-155 induction in response to *F.n.* requires the downstream activation of PI3K, ERK, and JNK.

NFκB is a critical mediator of TLR signaling as well as a myriad of other cellular responses [28], [29]. However, its role in miR-155 induction is unclear with one report showing that it was essential [30] but another concluding that it was not [27]. We surmised that its involvement could be dependent upon the nature of the stimulus, so we examined the role of NFκB within the context of *Francisella* infection. Here, PBM were pretreated with either the IκB kinase (IKK) inhibitor BAY7085 or with DMSO vehicle control for 90 minutes, followed by infection with *F.n.* at 50 MOI for 6 hours. As shown in Figure 3H, pretreatment with BAY7085 completely blocked the *Francisella*-induced miR-155 response. To verify the efficacy of this NFκB inhibition, TNFα secretion was measured after *Francisella* infection with or without pretreatment with BAY7085. As expected, there was no detectable TNFα release with BAY7085 pretreatment (Figure 3I). A lactate dehydrogenase (LDH) assay showed cytotoxicity of less than 10% after incubation with BAY7085 for 8 hours, again confirming that the cells were viable (data not shown). To confirm the requirement for NFκB, the peptide inhibitor SN50 was also used and found to block miR-155 induction (data not shown). Collectively, these results suggest that *Francisella*-induced miR-155 induction requires NFκB activation. This finding helps to explain why bacterial viability was required for maximal miR-155 induction (Figure 3A&B). A pervious report showed that maximal NFκB activation in response to *F.t.* LVS required bacterial viability and de novo bacterial protein synthesis [14]. Thus killed bacteria will have suboptimal NFκB activation as compared to live bacteria, and therefore suboptimal miR-155 induction.

### Bacterial Internalization and MiR-155 Induction

**Figure 4:**
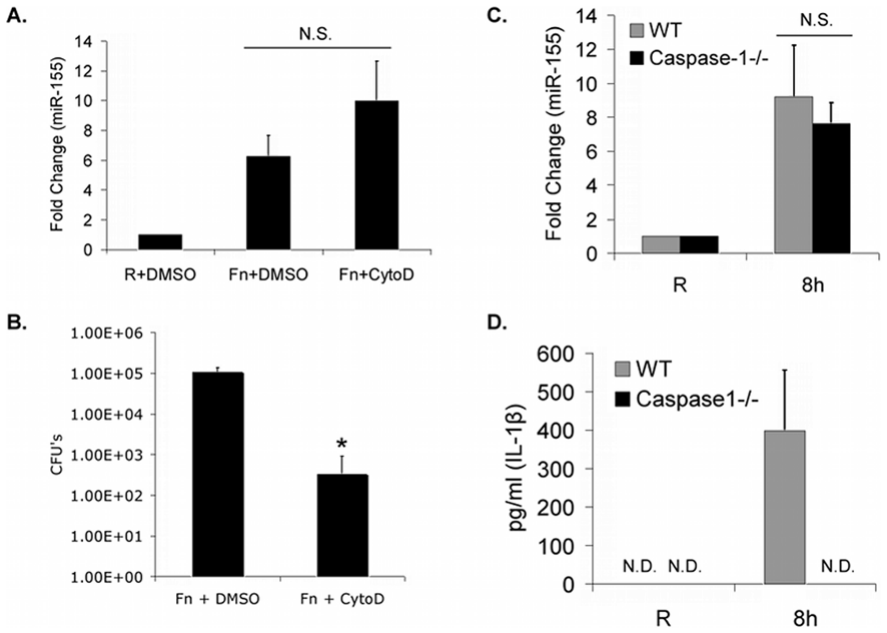
Effects of host-cell entry and inflammasome activation on miR-155 induction. (A) PBM were pretreated for 30 minutes with 5 µg/ml cytochalasin D (CytoD), infected with *F.n.* at an MOI of 50 for 6 hours, then relative miR-155 expression measured by qRT-PCR. (B) CFU assays were conducted in parallel with the samples from Figure 4A. (C) Wild-type (WT) and caspase-1^−/−^ bone marrow-derived macrophages were infected with *F.n.* at an MOI of 50 for 8 hours and miR-155 was assayed by qRT-PCR. (D) ELISAs were done to measure IL-1β in supernatants from the samples in Figure 4C. Graphs represent the mean and standard deviation from three independent infections. Data were analyzed by a paired Student *t* test. An asterisk (*) indicates a *p*-value<0.05.

Having found that TLR activity through MyD88 was critical for miR-155 induction, we hypothesized that cell surface contact with the bacteria would be sufficient to elicit miR-155. To test this, we pretreated PBM with an actin polymerization inhibitor, cytochalasin-D, or DMSO vehicle control for 30 minutes [31]. PBM were then infected with an MOI of 50 for 6 hours and miR-155 expression was measured by qRT-PCR (Figure 4A). MiR-155 expression was measured at 6 hours post-infection since the effectiveness of cytochalasin-D is reduced at later time points. MiR-155 induction in infected cells was not significantly different between vehicle control and cytochalasin-D treatments, suggesting that phagocytosis and internalization of *Francisella* may not be required during the early stages of infection for a miR-155 response. To verify the effectiveness of cytochalasin-D, CFU assays were performed in parallel with the same samples. After removal of viable extracellular bacteria with gentamicin, host cell lysates were plated and bacteria counted. Results showed approximately a 3-log reduction in uptake following cytochalasin-D pretreatment (Figure 4B).

### *F.n.*-Mediated MiR-155 Induction Is Independent of Caspase-1 Inflammasome Activation

One hallmark of *Francisella* infection is that the bacteria can escape from the phagosome into the cytosol within an hour after internalization [3], [32], which leads to inflammasome and caspase-1 activation as well as IL-1β release [33], [34]. To test whether inflammasome activation was required for the induction of miR-155, we infected wild-type or caspase-1 knockout bone marrow-derived macrophages with *F.n.* at an MOI of 50 for 8 hours. MiR-155 expression was measured by qRT-PCR and it was found that both the wild-type and caspase-1^−/−^ macrophages responded nearly identical (Figure 4C). Thus, *F.n.*-induced miR-155 expression is caspase-1-independent, suggesting that the caspase-1 inflammasome is not required. To verify lack of caspase-1 function in these BMM, IL-1β release was measured. Results (Figure 4D) showed that, as expected, IL-1β secretion by caspase-1^−/−^macrophages was impaired.

### MiR-155 Promotes Pro-Inflammatory Cytokine Production during *F.n.* Infection

**Figure 5:**
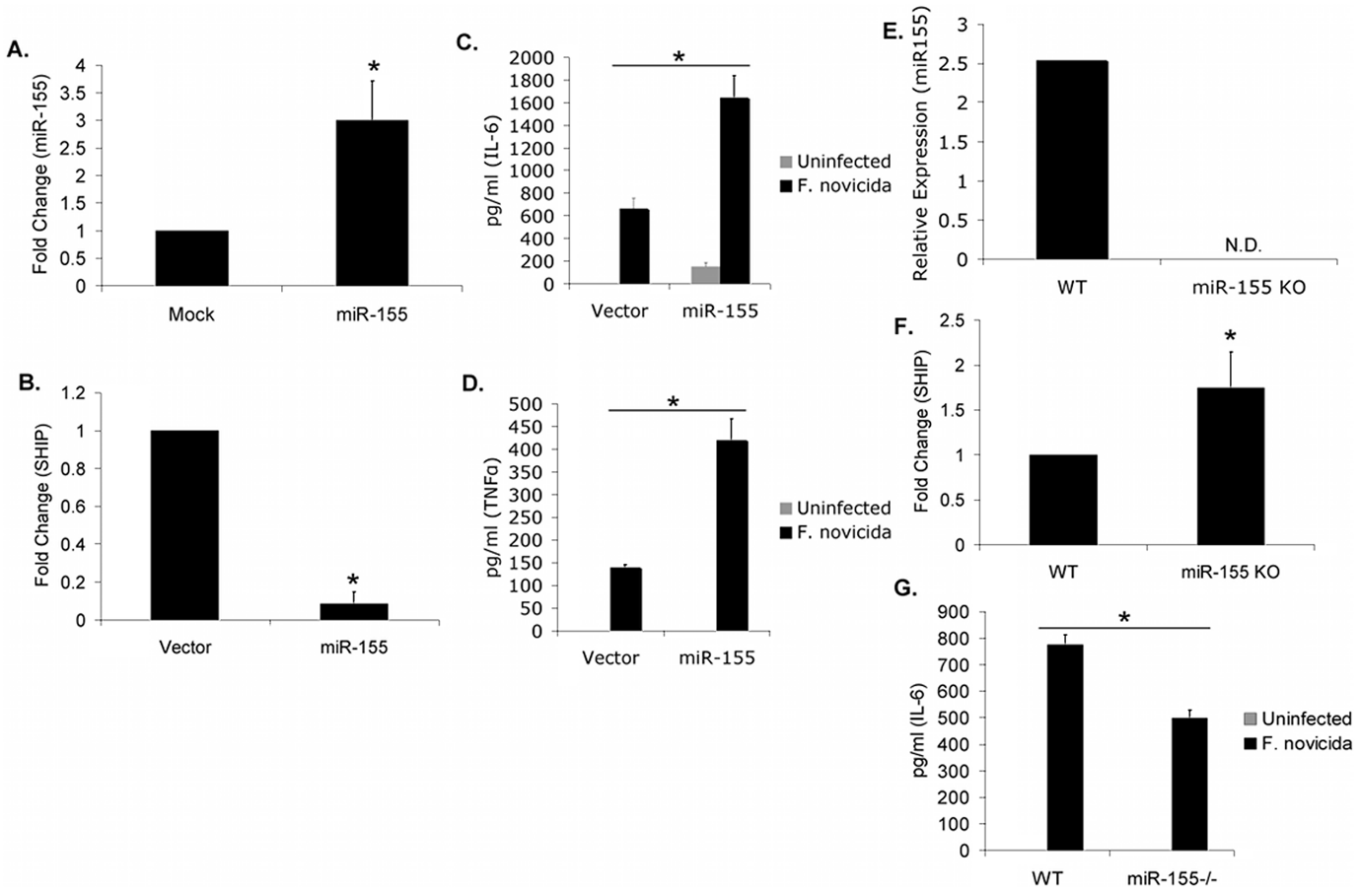
miR-155 promotes pro-inflammatory cytokine production during *F.n.* infection. (A) PBM were transfected with vector control or miR-155 overexpression construct. 22 hours post-transfection, RNA was extracted and miR-155 over-expression was verified by qRT-PCR. (B) SHIP expression was assayed from the samples in Figure 5A. (C–D) 14 hours post-transfection PBM were infected with *F.n.* at an MOI of 50 for 8 hours and (C) TNFα and (D) IL-6 measured in supernatants. (E) Wild-type and miR-155^−/−^ macrophages were assayed for miR-155 expression. (F) SHIP expression was assayed by qRT-PCR from the samples in Figure 5E. (G) Wild-type and miR-155^−/−^ macrophages were infected with *F.n.* at an MOI of 50 for eight hours. IL-6 in the supernatant was measured by ELISA. All experiments were performed in triplicate. Graphs represent the mean and standard deviation of samples from three independent experiments. Data were analyzed by a paired Student *t* test. An asterisk (*) indicates a *p*-value<0.05.

Activation of the PI3K/Akt pathway promotes the pro-inflammatory response to *F.n.*, and SHIP is an established negative regulator of this pathway [35]. We have previously demonstrated that SHIP^−/−^ macrophages display enhanced pro-inflammatory cytokine production in comparison to wild-type macrophages infected with *F.n.* [17]. Given the observations that miR-155 is strongly induced in response *F.n.* infection and that miR-155 can negatively regulate SHIP expression, we hypothesized that miR-155 exerts its pro-inflammatory function against *Francisella* by repressing SHIP. To test the functional consequence of miR-155 expression, PBM were transfected with either exon 3 of *BIC* (encoding the mature miR-155) or an empty vector. RNA was extracted 22 hours after transfection and qRT-PCR for miR-155 expression was performed. Over-expression resulted in an approximately 3-fold increase in miR-155 compared to vector-only transfection (Figure 5A). SHIP mRNA was then measured in the same samples by qRT-PCR, and results showed that miR-155 over-expression alone was sufficient to significantly decrease SHIP mRNA (Figure 5B). To determine the functional consequence for *F.n.* infection, PBM were infected 14 hours after transfection with *F.n.* at an MOI of 50 for 8 hours and the secretion of TNFα (Figure 5C) and IL-6 (Figure 5D) quantified by ELISA. Levels of both cytokines were significantly enhanced in PBM over-expressing miR-155 compared to those with vector control. Results from this over-expression of miR-155 are reflective of the results seen with SHIP^−/−^ macrophages infected with *F.n.* by Parsa *et al*. Collectively, these results are consistent with the hypothesis that miR-155 promotes pro-inflammatory cytokine production largely by downregulating SHIP.

To complement the data obtained from miR-155 over-expression, we tested the function of miR-155 in wild-type versus miR-155^−/−^ macrophages. The loss of miR-155 was verified by qRT-PCR (Figure 5E). Given that miR-155 targets SHIP, we examined SHIP mRNA expression in wild-type and miR-155^−/−^ macrophages. The loss of miR-155 was sufficient to lead to enhanced SHIP mRNA expression as compared to wild-type macrophages, further supporting the finding that miR-155 targets SHIP (Figure 5F). We then tested the pro-inflammatory response between wild-type and miR-155^−/−^ macrophages infected with *F.n.* MiR-155^−/−^ macrophages had significantly lower IL-6 production as compared to wild-type macrophages (Figure 5G). Collectively these data show that miR-155 negatively regulates SHIP and promotes inflammatory cytokine response to *F.n.*

### Virulent *F.t.* Induces Suboptimal MiR-155 and Pro-Inflammatory Cytokine Responses

**Figure 6:**
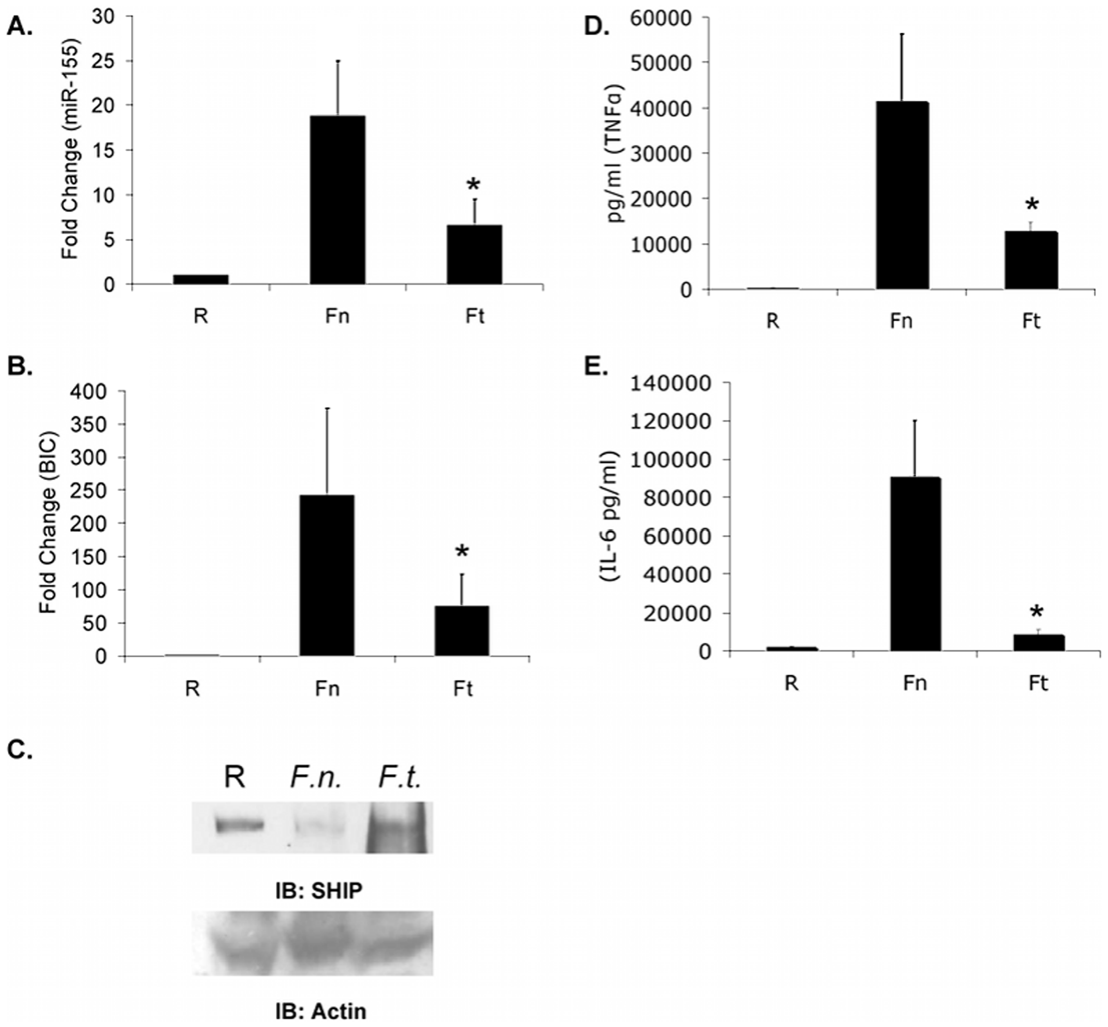
Virulent *F.t.* elicits suboptimal miR-155 and pro-inflammatory cytokine responses. (A–B) PBM (n = 7) were infected with *F.n.* or *F.t.* at an MOI of 100 for 24 hours. Relative miR-155 (A) and *BIC* (B) expression were measured by qRT-PCR. (C) PBM were infected with *F.n.* or *F.t.* at an MOI of 50 for 24 hours, then cell lysates probed for SHIP by Western blotting (top panel). The lower panel is a reprobe of the same membrane with anti-actin antibody. (D–E) PBM were infected with *F.n.* or *F.t.* at an MOI of 50 for 24 hours. TNFα (D) and (E) IL-6 in supernatants were measured by ELISA. Graphs represent the mean and standard deviation of samples from three independent infections. Data were analyzed by a paired Student *t* test. An asterisk (*) indicates a *p*-value<0.05.

Since we have established that the less virulent *F.n.* induces robust miR-155 induction in monocytes/macrophages and that it can regulate TNFα and IL-6, we next examined whether the miR-155 response was similar with the highly virulent *F.t.* isolate SCHU S4. PBM were infected with *F.n.* or *F.t.* for 24 hours. Relative expression of miR-155 (Figure 6A) and *BIC* (Figure 6B) were determined by qRT-PCR. While *F.t.* SCHU S4 led to modest miR-155 and *BIC* expression, it was significantly lower than that elicited by *F.n.*

Since miR-155 negatively regulates SHIP, and miR-155 induction is minimal following *F.t.* infection, we tested the ability of *F.t.* to down-regulate SHIP expression. We infected PBM with *F.n.* or *F.t.* for 24 hours. *F.n.* elicited a stronger miR-155 induction than *F.t.* and consistent with this, SHIP down-regulation was seen with infection by *F.n.* but not by *F.t.* (Figure 6C).

In light of these findings, we examined the production of TNFα and IL-6 in response to *F.n.* and *F.t.*, which we have now shown to be regulated by miR-155 and thus should be produced at lower levels in response to *F.t.* than to *F.n.* PBM were infected at an MOI of 50 for 24 hours and the levels of TNFα (Figure 6D) and IL-6 (Figure 6E) were determined by ELISA. Concordantly, it was found that the production of both cytokines was significantly lower in *F.t.*-infected samples than *F.n.*-infected samples. It is well established that *Francisella* bypasses or subverts host responses [11], [12], and the lack of miR-155 response after infection with the virulent strain may be a key contributing factor.

**Figure 7:**
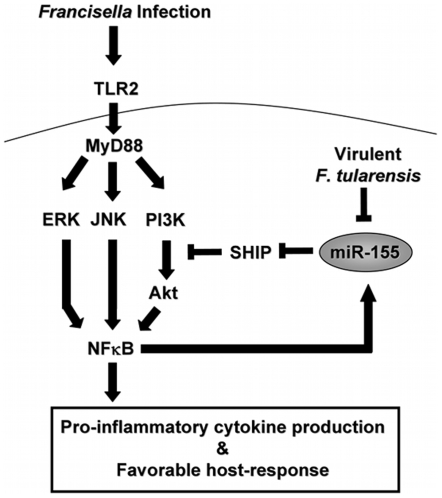
Model of miR-155 induction and function during *Francisella* infection. *Francisella* is recognized on the host cell surface by TLR2. The signal is transmitted through the adaptor protein MyD88. Subsequently MAPKs, PI3K and Akt are activated, which leads to enhanced NFκB activity, inflammatory cytokine production, and effective host response. SHIP negatively regulates the activation of Akt to prevent effective host response. During *Francisella* infection miR-155 is induced through the TLR signaling pathway, PI3K/Akt, ERK, JNK, and NFκB. MiR-155 induction in turn down-regulates SHIP to promote the activation of the PI3K/Akt pathway and inflammatory cytokine production. The highly virulent *F. tularensis* suppresses or subverts the induction of miR-155 in human monocytes, while the relatively avirulent *F. novicida* does not.

In summary we find that miR-155 is strongly induced *in vitro* and *in vivo* by *F.n.* through the TLR signaling pathway and NFκB activation; however, this does not require activation of the inflammasome. Intriguingly we found that the highly virulent *F.t.* leads to minimal induction of miR-155 as well as a lack of ability to downregulate SHIP expression and lower pro-inflammatory cytokine production than *F.n.*, which may contribute to the highly virulent nature of *F.t.* (Figure 7).

## Discussion

In this study we demonstrated that miR-155 is highly induced by *F.n.* infection and that it targets SHIP to regulate the immune response against this pathogen. Specifically we have found a novel role for miR-155 as a positive regulator of pro-inflammatory cytokine response to *Francisella*. Over a short period of time, miR-155 has emerged as an important player in B-cell lymphomas [36], immune response [24], and other functions [26], [37], [38]. The earliest studies of pathogen-induced miR-155 expression examined the avian leukosis virus (ALV). It was found that ALV induced high levels of *BIC* expression in infected chickens and that this was highly correlated with B-cell lymphoma [39], [40]. However, the most intriguing relationship between miR-155 and viruses is that both the Kaposi's-scarcoma-associated herpes virus [41] and the oncogenic Marek's disease virus [42] encode functional orthologs of miR-155. This suggests that miR-155 expression may actually benefit certain viruses, and one report has supported a role for miR-155 induction to maintain latent viral genomes through the negative regulation of an established miR-155 target, IKKε [43].

Deletion of miR-155 leads to system-wide changes in immune function. For example, B and T cell responses are compromised in mice genetically deleted for miR-155 [44], [45]. MiR-155^−/−^ mice vaccinated with tetanus toxin fragment C showed overall reduced IgM and switched antigen-specific antibody production from defects in B-cell function. MiR-155^−/−^ T cells also showed impaired IL-2 and IFNγ production after immunization. The T cells also exhibited a bias toward a T_H_2 response, possibly due to the targeting of *c-Maf*. Antigen presentation is also affected, as miR-155^−/−^ dendritic cells were less able to activate T cells expressing a transgenic receptor for ovalbumin in the presence of that protein. Thus, miR-155 serves an essential role in multiple immune cell types.

Here, we examined the role of miR-155 in monocytes and macrophages within the context of host response to bacterial pathogens. Previous studies demonstrated that PI3K/Akt pathway is host-protective against *Francisella* infection [18] and the finding that SHIP negatively regulates this pathway [17] led us to study the intricacies of SHIP regulation during *Francsiella* infection. As the currently-known regulators of SHIP did not seem to account for all of the changes seen in SHIP expression [46], we examined the possibility that microRNAs may play a role. We found that monocytes and macrophages express miR-155 in response to *Francisella* infection and that this down-regulated SHIP. During preparation of this manuscript it was reported by O'Connell et al. that miR-155 can directly target SHIP [21]. Our data are consistent with their findings, so the importance of miR-155 induction in response to *Francisella* is well supported.

Multiple TLR ligands are capable of inducing miR-155 [24], so it is logical that miR-155 induction in response to *F.n.* requires TLR2 and MyD88. This finding is of considerable interest because while there are additional receptors that are engaged and are of importance in the host response to *Francisella*, such as the complement receptor and mannose receptor [47]–[49], it appears that MyD88 and the TLR pathway is of critical importance for miR-155 induction. Downstream of MyD88 activation we found that ERK and JNK were partly required for miR-155 induction, which supports the finding by Yiu et al that found ERK- and JNK-dependent BCR induction of miR-155. In contrast to Yiu et al. but in agreement with Gatto et al. 2008, who used a viral protein to induce miR-155 in B-cells, we found that activation of NFκB was absolutely required for miR-155 induction by *Francisella*. It is then fitting that inhibition of PI3K blocked miR-155 induction, as PI3K controls NFκB activation in response to *Francisella*. Hence, it appears as though cell type, cellular context and/or the nature of stimulus may affect the intracellular machinery involved with miR-155 expression.

TNFα and IFNβ are at least two cytokines produced during *Francisella* infection that may contribute to miR-155 induction [24]. However, it has already been shown that these cytokines work through the TNFα receptor, while direct TLR stimulation can induce miR-155 independently of TNFR [24]. This is likely the case with *Francisella*, as it has been shown to activate TLR2 [14]. Cytosolic sensing of *Francisella* by the inflammasome triggers multiple host response events [33], [34]. Given that neither bacterial internalization nor caspase-1 was clearly required for miR-155 induction, we are lead to conclude that the cytosolic sensing of *Francisella* is of marginal importance and that the extracellular sensing via the TLR pathway is the driving force in miR-155 induction.

It is important to note that *F.t.* has been well established to inhibit host immune response. It has been shown in the mouse that *F.t.* does not elicit any inflammatory cytokine production from dendritic cells [12]. We have previously found that *F.t.* does elicit some inflammatory cytokine production from monocytes, but to a far lesser degree than *F.n.* [11]. Just recently it has been reported that the difference in the ability of *F.t.* to induce inflammatory cytokine production between human dendritic cells and monocytes is due to differences in CD14 expression on these cells. So that monocytes which have high CD14 expression are capable of eliciting some response whereas dendritic cells are not [50]. In any case, the ability of host cells to mount an effective immune response to *F.t.* is impaired by its highly subversive nature.

Finding that the less virulent *F.n.* can strongly induce miR-155 and down-regulate SHIP whereas the highly virulent *F.t.* elicits a suboptimal miR-155 response and an inability to down-regulate SHIP makes miR-155 of compelling interest for studies of host response to microbial pathogens. We have recently examined the mechanisms by which *F.t.* subverts host response [11] and found that it preferentially down-regulates both TLR2 and components of the PI3K/Akt pathway, whereas *F.n.* does not. This suggests a mechanism by which *F.t.* may prevent miR-155 induction, as TLR2 and PI3K/Akt are components of the miR-155 induction pathway by *Francisella*.

In the current study we found that miR-155 positively regulates TNFα and IL-6 production in response to *Francisella*. Given that miR-155 has been shown to target the negative regulator SHIP and that SHIP negatively regulates NFκB activation and pro-inflammatory cytokine production, we conclude that miR-155 is pro-inflammatory within the context of *Francisella* infection. Indeed, SHIP has already been shown to negatively regulate neutrophil responses to TLR2 activation via peptidoglycan [51] and macrophage responses to *Francisella* infection [17]. Further, miR-155 over-expressing mice [52] develop a similar myeloproliferative phenotype to that of SHIP^−/−^ mice [53]. Hence, it appears that as with miR-155 expression itself, numerous specific factors can influence the nature of response to miR-155.

In conclusion, this report demonstrates that miR-155 is induced by *Francisella* and that the host response against this pathogen is enhanced through the targeting of SHIP by miR-155. Further, the differential induction of miR-155 by virulent versus less virulent *Francisella* subspecies suggests that differences in miR responses may at least partially explain the highly pathogenic nature of *F.t.* versus *F.n.* Further studies are required to determine the exact cause(s) of this differential response, such as whether it is completely attributable to TLR2 and PI3K/Akt regulation and if so, how these are down-regulated by the virulent *Francisella*. To our knowledge, this is the first study to describe differential regulation of miR-155 by two bacteria of the same subspecies. We find that miR-155 induction inversely correlates with virulence in human monocytes.

## Materials and Methods

### Cell and Reagents

THP-1 cells were obtained from the American Type Culture Collection (ATCC) (Manassas, VA) and cultured in RPMI-1640 (Gibco-BRL, Rockville, MD) supplemented with 5% heat-inactivated fetal bovine serum (FBS) (HyClone, Logan, UT), L-glutamine, penicillin (10,000 U/ml) and streptomycin (10,000 µg/ml) (Invitrogen, Carlsbad, CA). CHO cells were obtained from the ATCC and cultured as previously described by [26]. The BAY7085 IKK inhibitor and Sn50 NFκB peptide inhibitor were a generous gift from Dr. Denis Guttridge (The Ohio State University). LY294002 (20 µM), U0126 (2.5 µM) and SP00125 (5 µM) were obtained from Calbiochem (San Diego, CA). SB203580 (5 µM) and DMSO vehicle control (0.2%) was obtained from Sigma-Aldrich (St. Louis, MO). Cytochalasin-D was purchased from Biosource (Camarillo, CA) and used at a concentration of 5 µg/ml.

### Transgenic and Knockout Mice

The C57BL/6J wild-type and TLR2 signaling mutant mice (TLR2^tm1kir/J^) were obtained from The Jackson Laboratory (Bar Harbor, MA). Wild-type and miR-155^−/−^ (B6.129S7-Mirn155^tm1Brd^) mice were obtained from Mutant Mouse Regional Resource Centers (MMRRC) (Columbia, MO).

### Bone Marrow-Derived Macrophages

Mice were sacrificed and the femurs were removed. Bone marrow was flushed from the femurs and the cells were cultured in DMEM (Invitrogen, Carlsbad, CA) with 10% heat-inactivated FBS, 30% sterile filtered L-cell conditioned media, 0.1% β-mercaptoethanol (BioRad, Hercules, CA), and penicillin/streptomycin for six to seven days. The murine fibroblast cell line L929 was a generous gift from Dr. Stéphanie Seveau (The Ohio State University). L929 cells were grown to confluence in minimum essential media (Invitrogen, Carlsbad, CA) supplemented with 10% heat-inactivated FBS (HyClone, Logan, UT), non-essential amino acids, sodium pyruvate and penicillin/streptomycin (Invitrogen, Carlsbad, CA). Conditioned media from the L929 cells was collected, passed through a 0.2 µm filter and used to supplement the murine macrophage cultures. After 6 to 7 days of culture, BMM were washed 3 times in sterile PBS, scraped, then plated overnight in 12-well tissue culture plates. Purity of harvested macrophages was verified though CD11b^+^ staining by flow cytometry.

### Peripheral Blood Monocyte Isolation

Human peripheral blood monocytes (PBM) were isolated by centrifugation through a Ficoll gradient followed by CD14-positive Magnet-Assisted Cell Sorting (MACS, Miltenyi Biotec, Auburn, CA) according to manufacturer instructions as previously described [11]. Flow cytometry using CD14 antibody showed a minimum of 98% purity for each sample. PBM were maintained in RPMI-1640 containing 10% heat-inactivated FBS and L-glutamine.

### Bacterial Infections

All infections were conducted in 5% or 10% FBS-containing RPMI-1640 without antibiotic. *F. novicida* U112 (JSG1819) and *F. tularensis* subspecies *tularensis* (SCHU S4) were generously provided by Dr. John Gunn (OSU), and grown on Chocolate II agar plates (Becton, Dickinson and Company, Sparks, MD) at 37°C. SCHU S4 infections were conducted by CDC approved select agent users at The Ohio State University BSL3 Select Agent facility in accordance with the BSL3 bio-safety plan. All SCHU S4 strain infected samples and matched reference samples were decontaminated in accordance with approved protocols by the BSL3 advisory committee to ensure effective killing of microbes before removal from the facility. MOI was determined by optical density at 600 nm and verified by plating the inoculum overnight and counting CFU as done previously [11]. Heat-killed *F.t. novicida* were prepared by heating at 98°C for 10 minutes. Paraformaldehyde (PFA)-killed *F.n.* were prepared by treating with 4% PFA for 30 minutes and then washing with PBS three times to remove residual PFA. Treated bacterial suspensions were plated on chocolate II agar to ensure effective killing. Pulse-chase infections were done by infecting for 2 hours, removing the media, washing the cells with sterile PBS, and then incubating in media containing 50 µg/ml gentamicin (Invitrogen, Carlsbad, CA) for 30 minutes. Cells were then washed again and incubated in media containing 10 µg/ml gentamicin for the duration of the experiments.

### In Vivo Challenge with *F.n.*

Wild-type mice were injected intraperitoneally with either 200 CFU of *F.n.* or PBS in accordance with institutional animal use protocols. The infectious dose was verified by CFU assays. Mice were euthanized at 24, 48 or 72 hours post-infection. The liver, lungs, and spleen were harvested, passed though a 70 µm cell strainer (BD, Bedford, MA), centrifuged briefly and then resuspended in TRIzol® reagent (Invitrogen, Carlsbad, CA). RNA extraction was then performed according to manufacturer instructions (Invitrogen).

### ELISA Cytokine Measurements

Sandwich ELISAs were done for human TNFα using kits from R&D Systems (Minneapolis, MN) and for human IL-1β using kits from eBioscience (San Diego, CA) as done previously [17].

### Western Blot Analysis

Cells were lysed in TN1 buffer (50 mM Tris [pH 8.0], 10 nm EDTA, 10 M Na_4_P_2_O_7_, 10 nM NaF, 1% Triton-X 100, 125 nM NaCl, 10 nM Na_3_VO_4_, 10 µg/ml of both aprotinin and leupeptin. Proteins were electrophoretically separated on 10% acrylamide gels, tranferred to nitrocellulose membranes and then probed with rabbit polyclonal anti-mouse SHIP (generously provided by Dr. K. Mark Coggeshall) or anti-human SHIP (Upstate Cell Signaling Solution, Lake Placid, NY). Goat polyclonal antibody against actin was purchased from Santa Cruz Biotechnology (Santa Cruz, CA). Rabbit antibodies against pIKKα and NFκBp65 were purchased from Cell Signaling (Beverly, MA). Detections were performed using HRP-conjugated secondary antibodies followed by enhanced chemiluminescence (GE, Buckinghamshire, UK) as previously described [17].

### Real-Time PCR

Cells were lysed in TRIzol® reagent (Invitrogen, Carlsbad, CA) and RNA isolation was completed according to the manufacturer's instructions. 10 to 100 ng of total RNA were used for reverse transcription as measured by a ND-1000 spectrophotometer (Nanodrop, Wilmington, DE). cDNA was made using the TaqMan® MicroRNA Reverse Transcription Kit (Applied Biosystems, Foster City, CA) and the specific reverse transcription primers for hsa-miR-155, mmu-miR-155, and the housekeeping genes RNU44 and RNU48 (human), sno412 and sno202 (mouse) (Applied Biosystems, Foster City, CA). The corresponding PCR primers were used with TaqMan® Universal PCR Master Mix, No AmpErase® UNG (Applied Biosystems, Branchburg, NJ) using an ABI PRISM 7900HT Fast PCR system. cDNA for *BIC* mRNA expression was primed for reverse transcription with 0.8 nM of random hexamer (Applied Biosystems, Foster City, CA) and analyzed by qRT-PCR using custom Taqman probes spanning exons 2–3 (Hs01374570) (Applied Biosystems, Foster City, CA). Control reactions containing no reverse transcriptase and no cDNA template were also performed. Samples were measured in triplicate for each experiment, and each experiment was performed at least 3 times. Relative expression was calculated as 2̂^−(CT Target−CT Housekeeping Gene)^ and significance was determined by a paired Student *t* test [26]. To simplify data presentation, relative expression values were converted to fold change over uninfected.

### Transfection

5 µg of pcDNA3.1 vector with or without exon 3 of human *BIC* was used for each transfection with 10×10^6^ PBM. Amaxa solution T was used for electroporation using program Y-01 as previously described [18]. Infections were performed 14 hours post-transfection. Optimal miR-155 expression was found between 12 and 14 hours post-transfection by qRT-PCR analysis. Cell viability was monitored by trypan blue staining. At this time approximately 70% of the original PBM were recovered/viable and the numbers were comparable in vector versus miR-155 transfected cells.

### Construction of psiCHECK/INPP5D

A fragment encompassing the INPP5D 3′UTR was PCR-amplified using the forward primer (5′->AGC CCT CAG TGA GCT GCC ACT GAG TCG ->3′) and reverse primer (5′->GAG TGA GAA AGG CAC AAT TTA ATT GG->3′). This was subcloned into the PCR2.1 vector following the manufacturer's protocol (Invitrogen). Plasmid DNA was isolated from transformed colonies and verified by dideoxy chain termination sequencing. The fragment was removed from the PCR2.1 plasmid by digestion with *EcoRI* and blunt-end ligated into the psiCHECK vector downstream of the *f-luc* reporter gene. The final construct was verified by dideoxy chain termination sequencing.

### Luciferase Reporter Assay

Dual-luciferase reporter assays were done using the psiCHECK vector with or without the 3′ UTR of SHIP, cotransfected with 50 ng of the pRL-CMV *Renilla* luciferase vector (Promega, Madison, WI). CHO cells were transfected with 0 to 50 nM synthetic miR precursor of miR-155 or scrambled miR control (Ambion) and luciferase reporter vectors using lipofectamine 2000 (Invitrogen, Carlsbad, CA). 48 hours post transfection cells were lysed with passive lysis buffer (Promega) and dual-luciferase activity was assayed using a luminometer as previously described [26].
